# Endothelial Specific Deletion of Autotaxin Improves Stroke Outcomes

**DOI:** 10.3390/cells12030511

**Published:** 2023-02-03

**Authors:** Susmita Bhattarai, Utsab Subedi, Shrivats Manikandan, Sudha Sharma, Papori Sharma, Chloe Miller, Md Shenuarin Bhuiyan, Srivatsan Kidambi, Vassilis Aidinis, Hong Sun, Sumitra Miriyala, Manikandan Panchatcharam

**Affiliations:** 1 Department of Cellular Biology and Anatomy, Louisiana State University Health Sciences, Shreveport, LA 71103, USA; 2 Department of Pathology and Translational Pathobiology, Louisiana State University Health Sciences, Shreveport, LA 71103, USA; 3 Department of Chemical and Biomolecular Engineering, University of Nebraska, Lincoln, NB 68588, USA; 4 Biomedical Sciences Research Center Alexander Fleming, 16672 Athens, Greece

**Keywords:** autotaxin (ATX), lysophosphatidic acid (LPA), ischemic stroke, endothelium, permeability

## Abstract

Autotaxin (ATX) is an extracellular secretory enzyme (lysophospholipase D) that catalyzes the hydrolysis of lysophosphatidyl choline to lysophosphatidic acid (LPA). The ATX–LPA axis is a well-known pathological mediator of liver fibrosis, metastasis in cancer, pulmonary fibrosis, atherosclerosis, and neurodegenerative diseases. Additionally, it is believed that LPA may cause vascular permeability. In ischemic stroke, vascular permeability leading to hemorrhagic transformation is a major limitation for therapies and an obstacle to stroke management. Therefore, in this study, we generated an endothelial-specific ATX deletion in mice (ERT2 ATX^−/−^) to observe stroke outcomes in a mouse stroke model to analyze the role of endothelial ATX. The AR2 probe and Evans Blue staining were used to perform the ATX activity and vascular permeability assays, respectively. Laser speckle imaging was used to observe the cerebral blood flow following stroke. In this study, we observed that stroke outcomes were alleviated with the endothelial deletion of ATX. Permeability and infarct volume were reduced in ERT2 ATX^−/−^ mice compared to ischemia–reperfusion (I/R)-only mice. In addition, the cerebral blood flow was retained in ERT2 ATX^−/−^ compared to I/R mice. The outcomes in the stroke model are alleviated due to the limited LPA concentration, reduced ATX concentration, and ATX activity in ERT2 ATX^−/−^ mice. This study suggests that endothelial-specific ATX leads to increased LPA in the brain vasculature following ischemic–reperfusion and ultimately disrupts vascular permeability, resulting in adverse stroke outcomes.

## 1. Introduction

Autotaxin (ATX) is a secretory enzyme (lysophospholipase D) responsible for cellular functions such as cell migration, metastasis, proliferation, and angiogenesis [1]. The hydrolysis of lysophosphatidylcholine (LPC) mediated by ATX produces lysophosphatidic acid (LPA), which acts through its six G-protein-coupled receptors, LPA1-6, eliciting diverse cellular signaling [2]. LPA is an abundant bioactive lipid with versatile signaling both in development (vascular and nervous system development [3]) and under pathological conditions (neuropathic pain, cancer, neurodegenerative diseases [4], atherosclerosis, diabetes, fibrosis, rheumatoid arthritis [5], etc.). ATX is the primary producer of LPA in the body; heterozygous ATX mice have been shown to produce half the normal level of plasma LPA [1]. ATX is secreted as an active glycoprotein and is widely present in biological fluids [6], along with broad tissue distribution [7]. A high expression of ATX mRNA has been detected in the brain, ovaries, lungs, intestines, and kidneys [7]. ATX expression determines and regulates the concentration of LPA in tissue and body fluids, which are likely to be modified under disease conditions.

Under pathophysiological conditions, ATX causes fibrosis in pulmonary diseases [8,9], disruption in energy homeostasis in diabetes/obesity [10], metastasis in various cancers [11,12,13], endothelial permeability in neurovascular diseases [14,15,16], exacerbation of diseases in atherosclerosis [17], and cardiac inflammation during myocardial infarction [18]. ATX is regulated under pathological conditions through various signaling molecules such as NFκB [19], STAT3 [20,21], TNF-β [22], or IL-6 [11]. Earlier instances of physiological and pathological functions of ATX demonstrated in various diseases strongly support the concept of ATX as a useful and reliable therapeutic target [2]. Various inhibitors of ATX (PF8380 [23], HA130 [24], GWJ-23 [25], and GLPG1690 [26]) and LPA receptors (Ki16425 [27] and BrPLPA [28]) are under study and in clinical trials to inhibit the ATX–LPA–LPAR axis in various diseases [29]. However, a thorough understanding of the mechanistic or tissue-specific role of ATX is essential for the development of effective therapies and treatments.

It has been shown that ATX is essential for embryonic development and vessel formation, as ATX-deficient mice die at embryonic day 9.5 (E9.5) due to vascular defects [1]. To study the cell-specific role of ATX, we created tamoxifen-inducible endothelial-specific ATX mice. This mouse escapes embryonic lethality, as ATX is knocked out in adulthood. Previously, we have shown in vivo that the level of LPA was increased in the vasculature following ischemic stroke [15] and also in vitro that ATX was elevated with oxygen–glucose deprivation in mouse brain microvascular endothelial cells [30]. In this study, we aim to delineate the role of endothelial-specific ATX in stroke. Studying ischemic stroke in these mice will show that endothelial-specific ATX plays a major role in ischemic–reperfusion vascular permeability and stroke outcomes.

## 2. Materials and Methods

### 2.1. Ischemic Stroke Model (MCAO Model)

All animal studies were performed following the procedures and protocols approved by the Institutional Animal Care and Use Committee (IACUC) at LSU Health Sciences Center—Shreveport. Additionally, the procedures and protocols were in accordance with the National Institutes of Health guide for the care and use of laboratory animals. Three-month-old male C57BL/6J mice underwent ischemia–reperfusion (I/R) surgery or sham surgery. For ischemia–reperfusion (I/R) surgery for the stroke mouse model, we used the transient focal right middle cerebral artery occlusion (MCAO) mouse model, in which ischemia was performed for 90 min and reperfusion for 24 h. Silicone rubber-coated monofilament MCAO sutures from Doccol Corporation (602356PK10Re) were used to occlude the right middle cerebral artery. The right middle cerebral artery was accessed through the exposed external carotid artery using the Longa method [31]. This model was established to simulate a human stroke with reproducible infarcts [32]. Placement of the filament induces ischemia; removal of the filament after 90 min causes reperfusion, which was performed under 1.5–3% isoflurane. Following reperfusion, mice were kept on a heating pad and observed for 1 h after surgery. Carprofen (2 mg/kg) was given subcutaneously for pain relief post-surgery. Clean bedding and soft food were placed in the cage following surgery. Laser speckle contrast imaging was used to monitor the relative blood flow before and after MCAO. Mouse brain infarcts were assessed using TTC (triphenyl tetrazolium chloride, Sigma T8877, St. Louis, MO, USA) staining; 1.5 mm thick brain slices were incubated in a 2% TTC solution at 37 °C for 30 min and imaged to analyze the infarct volume using ImageJ software, Version 1.53t.

### 2.2. Generation of Inducible Endothelial-Specific ATX Knockout Mice (ERT2 ATX^−/−^)

Inducible ERT2-Cre mice [33] and ATX flox (ATX^fl/fl^) mice [3] were used for breeding. The generation of the ATX^fl/fl^ mice used in the study followed the Cre/LoxP system approach, as reported previously [3]. For inducible endothelial deletion, mice having both the endothelial-specific receptor tyrosine kinase (*Tie2* promoter region) and the tamoxifen (TAM)-inducible estrogen receptor (ER) transgenic system were used; this generation of these mice has been reported previously [33]. PCR genotyping was performed to obtain inducible endothelial-specific ATX knockout mice (ERT2 ATX^−/−^). Three-month-old mice were injected with tamoxifen to activate the estrogen receptor.

### 2.3. Tamoxifen Treatment

Tamoxifen (2 mg/day) (Sigma T5648, St. Louis, MO, USA) prepared in corn oil was administered intraperitoneally for 5 consecutive days to obtain the endothelial-specific knockout of ATX in the presence of Cre recombinase. The ATX knockout mice were used for the I/R surgery after 2 days of the tamoxifen regime. Sham group mice were injected with corn oil only for the same duration.

### 2.4. ATX Activity Measurement

An analysis of ATX activity was performed as previously described in Bhattarai et al. [30]. Briefly, ATX activity was observed in mouse whole brain and brain slices using an AR-2 probe [34]. The AR2 probe (0.5 mg/kg) prepared in phosphate-buffered saline was injected retro-orbitally in mice 3 h before euthanization. After injection of the AR-2 probe, mice were perfused transcardially, and the brains were obtained. A near-infrared (NIR) imager LI-COR Odyssey (LI-COR Biosciences, Lincoln, NE, USA) was used to image the whole brain and 1.5 mm brain slices at 800 nm. The brain images were quantified and represented as pseudo-color images using Image Studio 5.2 software.

### 2.5. Evans Blue Permeability Measurement

The permeability in the mouse brains was measured as reported previously [30]. Evans Blue dye is a moderate fluorophore [35] with a fluorescence that can be measured at 700 nm using an NIR imager. An LI-COR Odyssey imager (NIR imager) was used for imaging the whole brain and 1.5 mm brain slices. The fluorescence intensity measured correlates directly with the amount of Evans Blue dye permeabilized in the brain tissue after the transcardial perfusion of mice. Mice were injected intraperitoneally 3 h before euthanization and brain isolation with 1.2 mL/kg 1% Evans Blue solution in phosphate-buffered saline. The fluorescence intensity measured was quantified and represented as a grayscale image using Image Studio software.

### 2.6. Cresyl Violet Staining

Cresyl Violet staining or Nissl staining was performed using Cresyl Violet dye to determine the neuronal loss in mouse brain cryosections mounted on slides [36]. After isolation, the mouse brains were fixed in 4% paraformaldehyde overnight and put through a series of 10%, 20%, and 30% sucrose solutions for 24 h in each solution at 4 °C. The brains were snap frozen using liquid nitrogen in an optimal cutting temperature (OCT) solution. Coronal sections 14 μm thick were cut using a cryotome. For Cresyl Violet staining, the mounted slices were dehydrated in an ascending series of ethanol solutions, followed by staining with Cresyl Violet acetate (Sigma, C5042) solution for 10 min. The slides were then dehydrated in an ascending series of ethanol solutions, followed by clearing with xylene and coverslipping with DPX Mounting medium (Sigma). Slides were examined using a microscope.

### 2.7. PCR Genotyping

Mouse genotyping was done using PCR primers A1 = CGCATTTGACAGGAATTCTT and C1 = ATCAAAATACTGGGGCTGCC for ATX FL and A1 = CGCATTTGACAGGAATTCTT and B2 = TACACAACACAGCCGTCTCA for the endothelial-specific ATX knockout band [3]. CRE1 = GCGGTCTGGCAGTAAAAACTATC and CRE2 = GTGAAACAGCATTGCTGTCACTT were used for genotyping Cre recombinase.

### 2.8. ELISA (Enzyme Linked Immunosorbent Assay)

An ATX ELISA kit (K-5600, Echelon Biosciences, Inc., Brisbane, Australia) and LPA ELISA kit (LS-F25111, LSBio, Seattle, WA, USA) were used according to the manufacturers’ instructions. ELISA blood plasma was used for both assays. The anticoagulant (buffered sodium citrate 3.2%) was at a ratio of 1:9 sodium citrate/blood for blood collection; the blood was centrifuged for 10 min to obtain the plasma supernatant. Plasma was diluted at 1:10 before use in the assays.

### 2.9. Immunostaining

Mouse brains were isolated after transcardial perfusion with saline, followed by 4% paraformaldehyde. Brains were cryosectioned as mentioned above in the Cresyl Violet staining section. For immunostaining, the brain slides were blocked using a blocking buffer (10% goat serum in phosphate-buffered saline and 0.2% Triton X-100) at room temperature. Following blocking, primary antibodies for LPA (Z-P200, Echelon Biosciences, Inc.), CD31 (557355, BD Biosciences, San Jose, CA, USA), and IBA1 (019-19741, Wako Chemicals, Richmond, VA, USA) were added overnight at 4 °C. Alexa fluor secondary antibodies (Thermo Fisher Scientific, Waltham, MA, USA) were added accordingly for 1 h at room temperature. Mouse brain slides were mounted using a Vecta Shield mounting medium with DAPI (H-1500, Vector Laboratories). Slides were then examined using a fluorescence microscope with a mounted Nikon camera.

### 2.10. Laser Speckle Imaging

Cerebral blood perfusion was performed in the experimental groups using a Perimed Laser Speckle Imager (Pericam PSI HR; Jakobsberg, Sweden). Following surgery and 24 h of reperfusion, mice were anesthetized using 3% isoflurane and oxygen while lying on a heating pad. Coronal skin was removed, and the skull of the mouse was exposed to capture the recordings. The camera of the imager was adjusted to 10 cm for all perfusion recordings. Cerebral blood flow in the right hemisphere of the mice was selected and analyzed. The cerebral blood flow perfusion data were measured and plotted as perfusion units.

### 2.11. Statistical Analysis

All statistical analyses were performed using GraphPad Prism Version 8 software. Data were reported as means ± standard error of the mean (SEM). One-way analysis of variance (ANOVA) followed by the Bonferroni’s post hoc test was performed for more than two groups. Student’s *t*-test (Mann–Whitney *U* test: nonparametric *t*-test) was performed for two groups. When comparing between groups, a *p*-value of <0.05 was considered significant.

## 3. Results

### 3.1. Generation and Genotyping of Inducible Endothelial ATX Knockout Mice (ERT2 ATX^−/−^)

**Figure 1:**
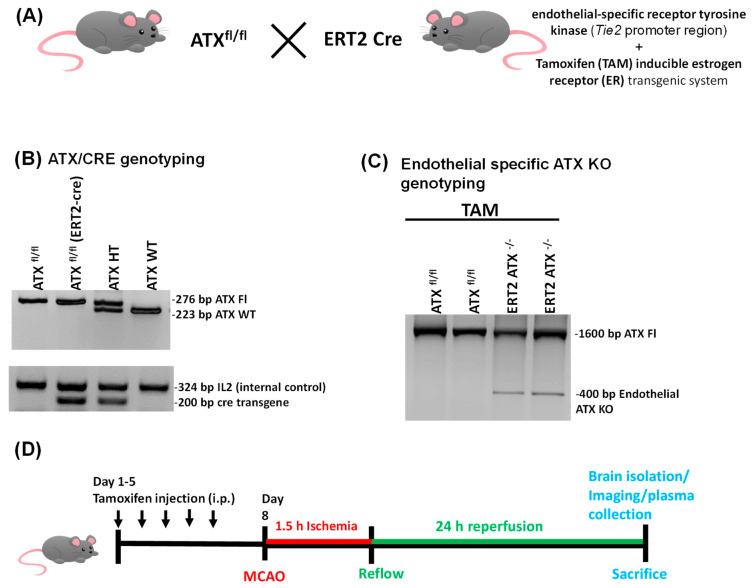
Generation and genotyping of endothelial-specific ATX knockout mice. (**A**) ATX flox (ATX ^fl/fl^) mice were bred with transgenic mice having an estrogen receptor-Cre fusion protein under the control of endothelial-specific receptor tyrosine kinase Tie2 promoter (ERT2 Cre mice) to generate tamoxifen (TAM)-inducible endothelial-specific ATX knockout mice. (**B**) DNA genotyping using primers for ATX and CRE to identify mice with Cre transgenic system plus ATX^fl/fl^ system to induce TAM-mediated knockout for ATX. (**C**) Mice brain tissue genotyping to show endothelial-specific ATX is knocked out after injection of TAM in ERT2 ATX^−/−^ mice. A band at 1600 bp shows the full ATX from other cells of the brain, and a 400 bp band is from the endothelial cells where ATX is knocked out after TAM injection. (**D**) Timeline representation of the experimental plan where mice were injected with tamoxifen for 5 days and then MCAO was performed in mice (1.5 h ischemia and 24 h reperfusion), followed by brain isolation for various experiments.

To generate endothelial-specific knockout of ATX in adult mice, ATX^fl/fl^ mice were bred with transgenic mice expressing a recombinant estrogen receptor with a Cre fusion protein under the control of the tyrosine kinase-Tie2 promoter (ERT2-cre mice) (Figure 1A). In these mice, the fusion protein Cre recombinase with mutated estrogen receptor will be active only in the presence of 4-hydroxy tamoxifen (TAM). Cre-positive ATX^fl/fl^ mice were injected with TAM at 3 months; Cre recombinase (under the control of the Tie2 promoter) is expressed only in the vascular endothelium, which produced endothelial-specific ATX knockout mice (ERT2 ATX^−/−^) (Figure 1A–D). DNA genotyping (Figure 1C) shows the endothelial-only ATX knockout band at 400 bp from the mouse whole brain tissue. For the experimental analysis, ATX^fl/fl^ and ERT2 ATX^−/−^ mice were used as described in the experimental plan outlined in Figure 1D. 

### 3.2. Inducible Inactivation of ATX in Adult Mice

**Figure 2:**
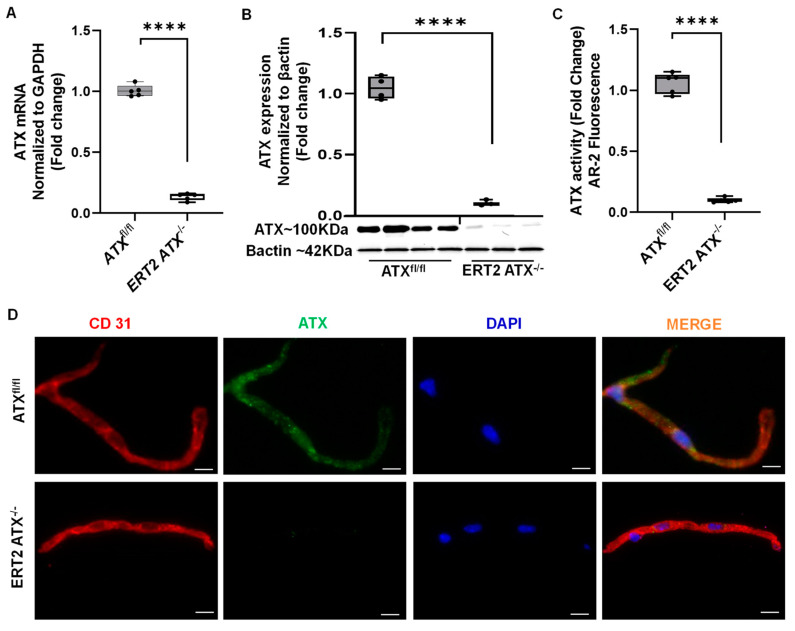
Tamoxifen-inducible Tie2-Cre-mediated inactivation of ATX lowers ATX expression in the brain microvasculature. (**A**) ATX mRNA expression was measured in brain microvascular tissue from ATX^fl/fl^ and ERT2 ATX^−/−^ mice and reported relative to the values in ATX^fl/fl^ in brain microvascular tissue (mean ± SEM from *n* = 5 animals per genotype). (**B**) Immunoblot analysis of ATX expression in brain microvascular tissue with actin used as a loading control. ATX expression was normalized to actin staining (*n* = 3–4 animals) and presented as mean ± SEM in arbitrary units, in which the density of ATX in the ATX^fl/fl^ samples was set to 1. (**C**) ATX activity determined in brain microvascular tissue using the AR-2 probe (*n* = 5 animals per genotype). Results are reported as the fold change in which the RFU of ATX in the ATX^fl/fl^ samples was set to 1 (mean ± SEM; *n* = 5 mice per genotype). (**D**) Representative images of immunostaining for CD31 (red) and ATX (green) in isolated brain microvascular tissue, whereas endothelial CD31 and ATX expression overlap in ATX^fl/fl^ brain microvascular tissue (magenta color) in ERT2 ATX^−/−^ mice. Scale bar = 100 μm.**** *p* < 0.0001 by *t*-test versus control.

The ATX mRNA levels were significantly downregulated in brain microvascular tissues of ERT2 ATX^−/−^ mice (Figure 2A). An immunoblot analysis of brain microvascular tissues indicated that the ATX levels were reduced in ERT2 ATX^−/−^ mice (Figure 2B), as was ATX activity measured in brain microvascular tissues (Figure 2C). Immunohistochemical analysis of brain microvascular tissues indicated that ATX was not present in endothelial cells (Figure 2D). 

### 3.3. Reduction in LPA Concentration in ERT2 ATX^−/−^ following I/R

**Figure 3:**
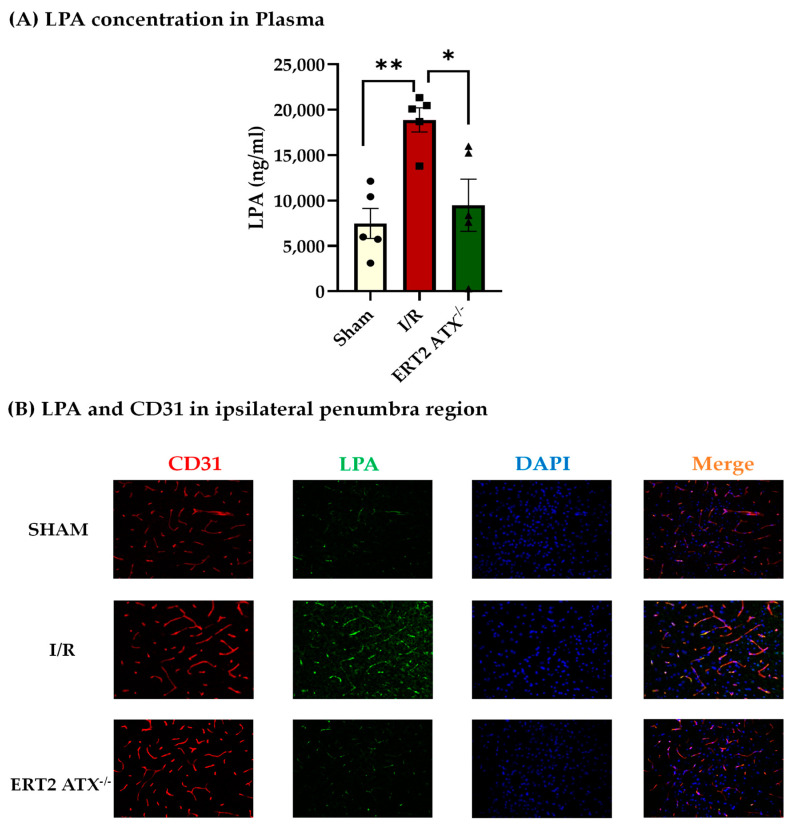
LPA decreased in ERT2 ATX^−/−^ following I/R. (**A**) LPA concentration in mice plasma quantified using ELISA kit in sham, I/R and ERT2 ATX^−/−^ mice, *n* = 5. (**B**) LPA (for lysophosphatidic acid) and CD31 (for endothelial cells/ blood vessels) immunostaining in mice brain cryosections, *n* = 5. LPA is labeled in green, CD31 (marker for endothelial cells) in red, and DAPI (marker for nuclei) in blue (20× magnification). All values are mean ± SEM. * *p* < 0.05 and ** *p* < 0.01 show significance compared to using ANOVA followed by the Bonferroni post hoc test.

As ATX produces bioactive lipid LPA through the hydrolysis of LPC, the LPA concentration was measured in mouse blood plasma after ischemia–reperfusion (I/R) for 1.5 h and 24 h, respectively, in all the following groups of mice: sham, I/R (ATX^fl/fl^ with ischemia–reperfusion), and ERT2 ATX^−/−^ (ERT2 ATX^−/−^ with ischemia–reperfusion). LPA measured using ELISA was elevated significantly in I/R mice as compared to sham mice but was significantly reduced in ERT2 ATX^−/−^ mice compared to I/R mice (Figure 3A). This suggests that the large amount of LPA produced during the ischemic and reperfusion insult in stroke pathology is contributed by the endothelial ATX. Mouse brain cryosections were also stained for the LPA antibody in all the groups. LPA was observed to be increased in brain tissue with I/R but was reduced with the endothelial deletion of ATX in ERT2 ATX^−/−^ (Figure 3B). These results suggest that endothelial ATX could be a major contributor to LPA production during pathologies. Generation and genotyping of inducible endothelial ATX knockout mice (ERT2 ATX^−/−^). 

### 3.4. ATX Concentration and Activity Reduced in ERT2 ATX^−/−^ following I/R

**Figure 4:**
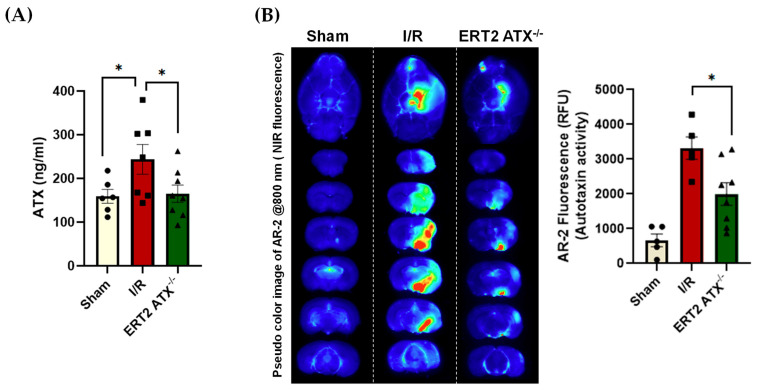
Reduced ATX and its activity in ERT2 ATX^−/−^ following I/R. (**A**) ATX concentration in mice plasma quantified using the ELISA kit in sham, I/R, and ERT2 ATX^−/−^ mice, *n* = 6–8. (**B**) Enzymatic activity for ATX measured using the AR-2 fluorescence probe and quantified as relative fluorescence units (RFU) in sham, I/R, and ERT2 ATX^−/−^ mice brains. Pseudo-color images of the AR-2 probe at 800 nm in an infrared imager are shown as representative images for ATX activity, *n* = 5–8. All values are mean ± SEM. * *p* < 0.05 shows significance compared using ANOVA followed by the Bonferroni post hoc test.

The ATX plasma concentration was measured in all groups (sham, I/R, and ERT2 ATX^−/−^) using ELISA (Figure 4A). The ATX concentration in I/R mice was significantly elevated compared to that of the sham mice, whereas, with the endothelial deletion of ATX, the concentration of ATX in plasma following an injury was significantly lower when compared with the I/R group (Figure 4A). Our group has shown that ATX is dispensable for adult life [37], and we observed no change in the plasma ATX and LPA levels in sham ERT2 ATX^−/−^ as compared to sham control mice (data not shown). As ATX is an enzyme, the enzymatic activity of ATX was quantified with an AR-2 probe, which is an analog of LPC. Imaging of the AR-2 probe in mouse brain tissue provided evidence that ATX activity was elevated in I/R mice compared to that of the sham mice (Figure 4B). The ATX activity observed and quantified in ERT2 ATX^−/−^ mice was found to be significantly lower compared to that of the I/R mice (Figure 4B). These results suggest that ATX activity is increased in stroke, and the endothelium could be the main contributor during ischemic–reperfusion injury. 

### 3.5. Infarct Size Reduced with Endothelial-Specific Deletion of ATX

**Figure 5:**
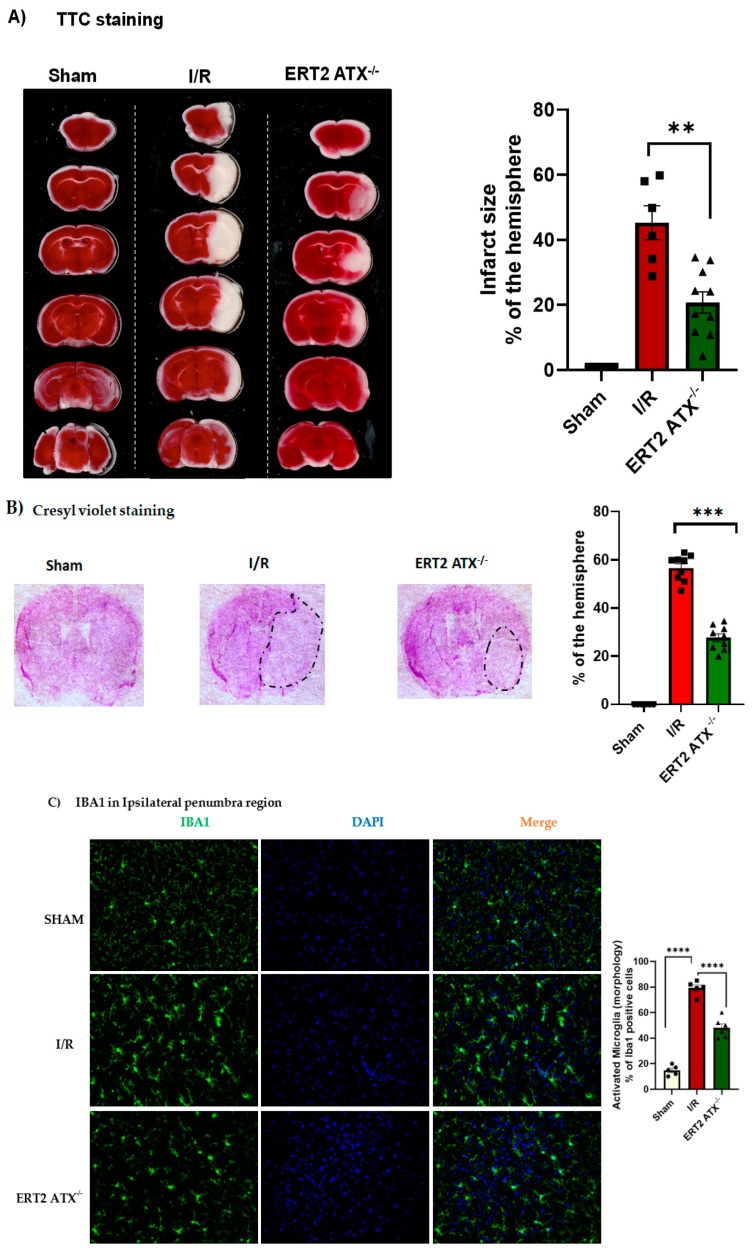
Infarct size post-injury decreased in ERT2 ATX^−/−^ mice. (**A**) TTC (triphenyl tetrazolium chloride) staining was performed in mice brain slices and quantified the infarct region as a % of the ipsilateral hemisphere in sham, I/R, and ERT2 ATX^−/−^ mice, *n* = 6–10. (**B**) Cresyl violet staining was performed in cryosectioned mice brain sections, and the infarct region was quantified as a % of the ipsilateral hemisphere in sham, I/R, and ERT2 ATX^−/−^ mice, *n* = 6–10. (**C**) Immunostaining for IBA1 (marker for microglia) and DAPI (marker for nuclei) was performed in cryosectioned mice brain sections in sham, I/R, and ERT2 ATX^−/−^ mice (20× magnification). Iba-1-positive cells were characterized morphologically according to their activation/resting state and expressed as a percent of their total number, *n* = 5–6. All values are mean ± SEM (*n* = 6–10). ** *p* < 0.01, *** *p* < 0.001, and **** *p* < 0.0001 compared to I/R using a Mann–Whitney *U* test.

Brain tissue damage following stroke was measured using TTC staining and Cresyl Violet staining. With TTC staining, the infarct size was observed to be significantly reduced in ERT2 ATX^−/−^ mice compared to that of the I/R mice (Figure 5A). With Cresyl Violet staining, a reduction in infarct size was also observed when endothelial ATX was deleted in ERT2 ATX^−/−^ (Figure 5B). Additionally, the Cresyl Violet staining data suggest lesser neuronal loss in ERT2 ATX^−/−^ mice compared to I/R mice (Figure 5B), as Cresyl Violet stains neuronal cells in the brain tissue. Reduction in the ATX and LPA levels during the pathological phases of a stroke could reduce the neuronal excitotoxicity and neuronal loss. Along with the decreased infarct size following injury, ERT2 ATX^−/−^ mice also showed lowered inflammation observed with reduced IBA1 microglial staining in the ipsilateral penumbral region (Figure 5C). Microglial numbers were quantified morphologically (ramified microglia as inactivated and amoeboid microglia as activated).

### 3.6. Improved Stroke Functional Outcomes in ERT2 ATX^−/−^ Mice

**Figure 6:**
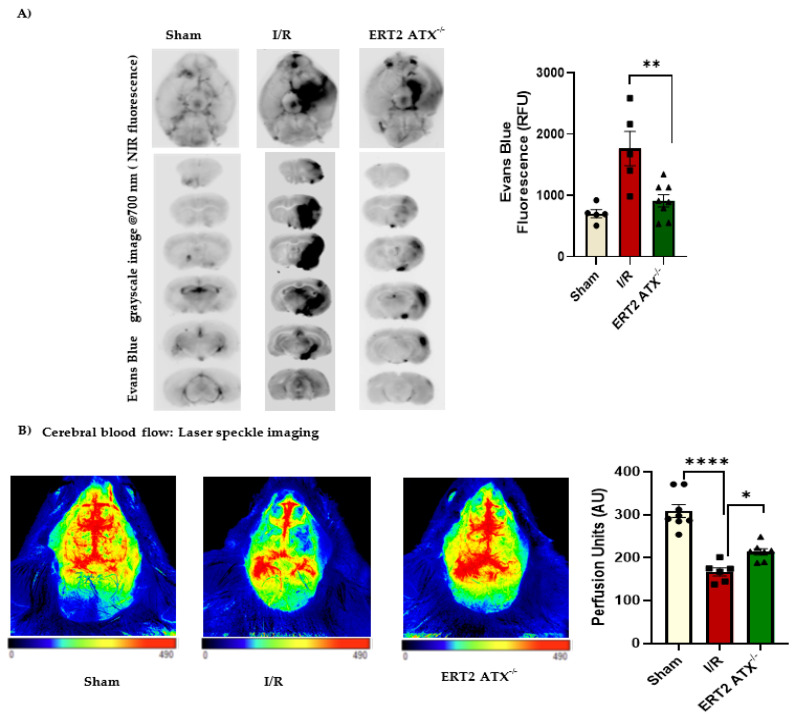
Better functional stroke outcomes observed in ERT2 ATX^−/−^ mice. (**A**) Vascular permeability measured using Evans blue fluorescence. Evans blue fluorescence obtained at 700 nm in the infrared imager represented as grayscale images and quantified as relative fluorescence units (RFU) in sham, I/R, and ERT2 ATX^−/−^ mice brains, *n* = 5–8. (**B**) Cerebral perfusion was measured with Laser Speckle imaging and representative images are shown for sham, I/R, and ERT2 ATX^−/−^ mice brains. Perfusion units as an arbitrary unit (AU) were used for quantification of cerebral blood flow in the ipsilateral hemisphere of the mice brains, *n* = 6–8. All values are mean ± SEM. * *p* < 0.05, ** *p* < 0.01, and **** *p* < 0.0001 show significance compared to using ANOVA followed by the Bonferroni post hoc test.

As it has been observed that the ATX–LPA axis contributes to the breakage of the BBB [15], it was essential to measure the permeability in all groups (sham, I/R, and ERT2 ATX^−/−^). Endothelial vascular permeability was measured in mouse brain tissue using Evans Blue dye. The results showed that permeability was increased with I/R, except in ERT2 ATX^−/−^ mice, where the Evans Blue permeability was significantly reduced (Figure 6A). Due to ablated endothelial ATX, the permeability was lower, suggesting that endothelial ATX contributes to BBB breakage during stroke pathological progression. Cerebral blood flow was measured in mice using laser speckle contrast imaging to quantify brain tissue blood perfusion. It was observed that perfusion was significantly lower in mice following I/R compared to sham, whereas, in ERT2 ATX^−/−^ mice, perfusion in the mouse brain tissue was significantly improved compared to I/R mice (Figure 6B). These results suggest that the deletion of ATX from endothelial cells improves brain functions such as vascular permeability and cerebral microcirculatory perfusion, emphasizing the undesirable role of endothelial ATX in vascular pathologies including stroke.

## 4. Discussion

The secretory enzyme ATX hydrolyzes extracellular LPC to LPA [38]. Acting via its G-protein receptors, extracellularly produced LPA aids in the pathological progression of various vascular and neurological diseases. In particular, LPA has been observed in BBB breakage in endothelial cells, resulting in vascular permeability [39,40,41]. A compromised or dysfunctional BBB is a major contributing factor in disease initiation and progression in neurovascular diseases such as brain tumors, epilepsy, multiple sclerosis, trauma, and stroke [42]. Elevated levels of LPA have been observed in the brain tissue microenvironment after stroke [43,44], suggesting that LPA plays a role in vascular permeability. Previous studies have shown that endothelial integrity is affected by LPA through the Rho kinase pathway [30,41,45]. Recognition of cell-specific production and the mechanism of action of the ATX–LPA axis is crucial to the development of targeted and effective therapy rather than relying on nonspecific signaling and the action of LPA. For vascular diseases such as stroke, it is essential to understand the major factors contributing to disease progression. Targeting the endothelium to prevent it from becoming dysfunctional is a reliable future therapeutic strategy [46,47,48] to limit the poor outcomes common in strokes. We generated a Tie2 promoter-driven deletion of ATX in adult mice in this study, resulting in ERT2 ATX^−/−^ mice. Cre recombinase in combination with the estrogen receptor transgenic system helped to produce an inducible endothelial deletion of ATX following the injection of TAM in these mice (Figure 1C). The findings in this study suggest that endothelial-specific ATX has a pathological effect on ischemic stroke. This pathological effect may occur as the result of increased levels of LPA in the system and in the vasculature, ultimately affecting the permeability of the brain following ischemic–reperfusion injury.

The effects of ATX have been shown to be systemic in diseases such as sepsis [49], obesity [50], idiopathic pulmonary fibrosis [51], chronic liver diseases [52], and in an autocrine [53,54]/paracrine manner in various cancers and rheumatoid arthritis [55,56]. Endothelial ATX may be both systemic and autocrine/paracrine. Apart from the action of endothelial ATX or LPA in the vasculature, the ATX/LPA produced by endothelial cells is also released into the circulation. In this study, we observed that, with an endothelial-specific knockout, ATX production and its release into the plasma was limited following injury in ERT2 ATX^−/−^ mice (Figure 4A). Additionally, ATX activity was significantly lowered in our mouse stroke model with the endothelial-specific deletion of ATX (Figure 4B). We observed that endothelial-specific ATX increased LPA production in the stroke microenvironment, as well as releasing it into the circulation. This suggests that the endothelial ATX–LPA axis could act in both a systemic and an autocrine/paracrine manner following ischemia–reperfusion.

The role of endothelial-specific ATX has also been studied in atherosclerosis, where ATX deletion in endothelial cells reduced atherosclerotic plaque. The plasma levels of different LPAs such as LPA 16:0, LPA 18:0, and LPA 18:1 were strongly correlated with the endothelial expression of ATX, suggesting that endothelial expression aids in atherosclerosis progression [57]. Similarly, we also observed that endothelial ATX contributes to stroke progression, specifically by producing LPA and increasing the permeability. We have shown that LPA in the brain disrupts vascular permeability by affecting BBB junctional proteins [15,30]. In this study, we observed an increase in the concentrations of plasma LPA (Figure 3A), as well as tissue LPA (Figure 3B), following stroke injury. The increase in LPA species following stroke was also observed by Ueda et al. [43]. However, in the endothelial-specific ATX knockout mouse model, the LPA concentration in plasma and tissue following injury was relieved, along with reduced permeability in these mice (Figure 6A), suggesting a strong correlation between injury-induced LPA production and permeability in stroke. These results provide evidence that the endothelial-specific induction of LPA may play a prominent role in disease exacerbation in ischemia–reperfusion.

Increased permeability following stroke limits the use of r-tPA as a therapeutic approach due to the increased risk of hemorrhagic transformation [58]. The alleviation of ischemic stroke could be achieved by targeting endothelial-specific ATX inhibitors before administrating r-tPA, increasing the range of patients eligible for r-tPA. Recently, the treatment regimens available to stroke patients have evolved. Acute ischemic stroke management is progressing, leaving behind the classical universal time window approach and focusing instead on tissue preservation, which can be accomplished by preserving the cerebral blood flow, managing the penumbra, and ultimately reducing the risk of a second event [59]. While advances have been made in modern stroke treatments, their benefit to the majority of patients is truly low due to therapeutic limitations. Novel treatment strategies are essential when using acute therapeutic interventions focused on vascular strengthening and neuroprotection [60]. In this study, we observed that, with a reduction in ATX concentration and activity following stroke, neuronal loss was also improved (Figure 5B), and microglial activation in the penumbra was lessened (Figure 5C), along with reduced vascular permeability (Figure 6A). Cerebral hemodynamics can be used to predict the severity of stroke, as well as the rate of recovery after therapeutic intervention [61,62]. Here, the functional dynamics (cerebral blood flow) were significantly improved; for example, the preservation of blood perfusion was observed when endothelial ATX was deleted, implying that ATX has a negative impact during the pathological progression of stroke.

New therapeutic paradigms for stroke can be achieved by combining new drugs, old drugs, and modern technologies. Using ATX inhibitors or targeted ATX inhibition may be an excellent approach to address this new concept of stroke management. In this study, we observed that ATX deletion from endothelial cells improved the infarct size, permeability, and cerebral blood flow. Apart from mortality, stroke is one of the leading causes of disability worldwide [63], so using ATX–LPA axis inhibitors as interventions or adjuvants could be helpful in stabilizing stroke patient conditions for better outcomes. A recent study by Bitar et al. also observed similar findings to this study that astrocyte-specific inhibition ATX was able to improve stroke outcomes, specifically neuronal excitability in mice [64]. These findings attest to the negative role played by ATX in stroke outcomes. ATX is indispensable for embryonic development [1] but is not required in adulthood [37], making ATX a promising and reliable target for the development of drugs to limit stroke outcomes.

## 5. Conclusions

In this study, we observed that ATX produced by the endothelium could be a major factor responsible for increases in the permeability and infarcts and adverse effects on the cerebral blood flow during cerebral ischemic–reperfusion. LPA produced specifically by endothelial cells in the vasculature can disrupt the BBB, leading ultimately to neuronal loss and glial activation. With the deletion of ATX from the endothelium, the microvasculature might be more resilient due to reduced LPA. We found evidence that the functional outcomes of ischemic stroke were alleviated with ATX deletion. Therefore, endothelial ATX can be targeted to make better and more effective therapies for stroke management.

## Data Availability

Not applicable.
